# Machine learning from *Pseudomonas aeruginosa* transcriptomes identifies independently modulated sets of genes associated with known transcriptional regulators

**DOI:** 10.1093/nar/gkac187

**Published:** 2022-03-31

**Authors:** Akanksha Rajput, Hannah Tsunemoto, Anand V Sastry, Richard Szubin, Kevin Rychel, Joseph Sugie, Joe Pogliano, Bernhard O Palsson

**Affiliations:** Department of Bioengineering, University of California, San Diego, La Jolla, USA; Division of Biological Sciences, University of California San Diego, La Jolla, CA 92093, USA; Department of Bioengineering, University of California, San Diego, La Jolla, USA; Department of Bioengineering, University of California, San Diego, La Jolla, USA; Department of Bioengineering, University of California, San Diego, La Jolla, USA; Division of Biological Sciences, University of California San Diego, La Jolla, CA 92093, USA; Division of Biological Sciences, University of California San Diego, La Jolla, CA 92093, USA; Department of Bioengineering, University of California, San Diego, La Jolla, USA; Department of Pediatrics, University of California, San Diego, La Jolla, CA, USA; Center for Microbiome Innovation, University of California San Diego, La Jolla, CA 92093, USA; Novo Nordisk Foundation Center for Biosustainability, Technical University of Denmark, Kemitorvet, Building 220, 2800 Kongens, Lyngby, Denmark

## Abstract

The transcriptional regulatory network (TRN) of *Pseudomonas aeruginosa* coordinates cellular processes in response to stimuli. We used 364 transcriptomes (281 publicly available + 83 in-house generated) to reconstruct the TRN of *P. aeruginos*a using independent component analysis. We identified 104 independently modulated sets of genes (iModulons) among which 81 reflect the effects of known transcriptional regulators. We identified iModulons that (i) play an important role in defining the genomic boundaries of biosynthetic gene clusters (BGCs), (ii) show increased expression of the BGCs and associated secretion systems in nutrient conditions that are important in cystic fibrosis, (iii) show the presence of a novel ribosomally synthesized and post-translationally modified peptide (RiPP) BGC which might have a role in *P. aeruginosa* virulence, (iv) exhibit interplay of amino acid metabolism regulation and central metabolism across different carbon sources and (v) clustered according to their activity changes to define iron and sulfur stimulons. Finally, we compared the identified iModulons of *P. aeruginosa* with those previously described in *Escherichia coli* to observe conserved regulons across two Gram-negative species. This comprehensive TRN framework encompasses the majority of the transcriptional regulatory machinery in *P. aeruginosa*, and thus should prove foundational for future research into its physiological functions.

## INTRODUCTION

*Pseudomonas aeruginosa* is an opportunistic pathogen that can be found in diverse environments such as soil, water, plants and humans (1,2). It is one of the major causative agents of hospital-acquired nosocomial infections and the leading cause of lung infection in people suffering from cystic fibrosis (CF) (3,4). All major biological processes in *P. aeruginosa* are controlled by a complex transcriptional regulatory network (TRN) that is yet to be fully elucidated. TRNs constitute the underlying framework for understanding the developmental and physiological responses of organisms, and define the relationships between transcription factors (TFs) and their target genes in response to diverse stimuli (5,6). Knowledge of the TRN of *P. aeruginosa* and other pathogenic bacteria would be beneficial in elucidating novel drug targets, understanding the functions of their various virulence factors (6), as well as important for designing new or combinatorial therapies against *P. aeruginosa* infections. Today, machine learning approaches, such as independent component analysis (ICA), can be applied to sufficiently large transcriptomic datasets to identify independent signals in the data, which can then be annotated with mechanistic data to improve our understanding of transcriptional regulation in bacteria (7).

ICA is a decomposition method to separate the multivariate signals into independent signals and their relative strengths. A study of 42 TRN inference methods, which included clustering, network inference and other decomposition methods, demonstrated that decomposition methods based on ICA were the best at recapitulating known regulatory modules (8). ICA used to identify independent signals in complex data sets (9), has been applied to data sets of bacterial transcriptomes to identify independently modulated sets of genes called iModulons and the transcriptional regulators that control them (10–12). iModulons have been used to study the adaptive evolution trade-off during oxidative stress under naphthoquinone-based aerobic respiration (13), mutations in the OxyR transcription factor and regulation of the ROS response (14), and the host response to expression of heterologous proteins (15). We have also used ICA to elucidate quantitative TRN structures of *Staphylococcus aureus* (10), *Escherichia coli* (11) and *Bacillus subtilis* (12), which are presented in interactive dashboards on the iModulonDB.org website (16). ICA-based methods were also used to classify the tumor samples (17,18) and the connection of identified transcriptional modules to the diseased state (19).

In this study, we applied ICA to high-quality RNA-seq expression profiles of *P. aeruginosa* to decipher the overall structure of its TRN, expanding upon the current understanding of its regulatory networks (20–22). We incorporated in-house generated RNA-seq data from diverse conditions such as osmotic stress, low pH, oxidative stress, and micronutrient supplementation, and integrated all publicly available data of sufficient quality from the NCBI Sequence Read Archive as of October 20, 2020 (23). We assembled the largest RNA-seq compendium for *P. aeruginosa* to-date, and used ICA to reveal the relationship between iModulon activities and specific stimuli.

iModulons can use co-expression patterns to define the functional gene composition of a biosynthetic gene cluster (BGC), since genes in biosynthetic pathways are usually co-expressed. BGCs are clusters of genes that synthesize specialized secondary metabolites (24), such as pyochelin, pyoverdine, pyocyanin and bacteriocins (25–28). These specialized metabolites are of particular interest because of their diverse range of functions, and they contribute to the ability of *Pseudomonas* to survive in different environments, including the human lung (29). The comprehensive antiSMASH software uses sequence comparison to detect BGCs, but assigns BGC borders that were empirically determined and defined in the detection rules (30). iModulons are able to capture genes regulated by the same regulator, which makes them an accurate and efficient way to define the genomic boundaries without needing to generate specific gene knockouts; thus, iModulons can assist in annotating the BGCs and their accessory functions. The TRN structure established here represents a significant advance toward understanding the complex transcriptional regulation of *P. aeruginosa* under different growth conditions. Further, our study identifies several hypotheses from the transcriptomic data that are relevant to *P. aeruginosa* infections.

## MATERIALS AND METHODS

### RNA extraction and library preparation

The *P. aeruginosa* PAO1 and PAO1(*ΔmexB*) strains were used in this study. We extracted RNA samples for 25 unique conditions including different media types (M9, CAMHB, LB, RPMI + 10% LB), oxidative stress (treatment with paraquat), iron starvation (treatment with DPD), osmotics stress (high NaCl), low pH, various carbon sources (succinate, glycerol, pyruvate, fructose, sucrose, *N*-acetyl glucosamine), micronutrients (copper, iron, zinc, sodium hypochlorite). All conditions were collected in biological duplicates and untreated controls were also collected for each set to rule out the possibility of the batch effect (Supplementary Notes 1).

In brief, strains were grown overnight at 37°C, with rolling, in appropriate media types for the testing condition of choice. Overnight cultures were then diluted to a starting OD_600_ of ∼0.01 and grown at 37°C, with stirring. Once cultures reached the desired OD_600_ of 0.4, 2 ml cultures were immediately added to centrifuge tubes containing 4 mL RNAprotect Bacteria Reagent (Qiagen), vortexed for 5 s and incubated at room temperature for 5 min. Samples were then centrifuged for 10 min at 5000 × g and the supernatant was removed prior to storage at −80°C until further processing. In conditions involving antibiotic treatment, when the bacterial culture had reached an OD_600_ of ∼0.2, antibiotics were added at 2× or 5× their MIC in the appropriate media type and allowed to incubate at 37°C, with stirring, for an additional hour prior to sample collection.

Total RNA was isolated and purified using a Zymo Research Quick-RNA Fungal/Bacterial Microprep Kit from frozen cell pellets previously harvested using Qiagen RNAprotect Bacteria Reagent according to the manufacturers' protocols. Ribosomal RNA was removed from 1 ug Total RNA with the use of a thermostable RNase H (Hybridase) and short DNA oligos complementary to the ribosomal RNA, performed at 65°C to prevent non-specific degradation of mRNA. The resulting rRNA-subtracted RNA was made into libraries with a KAPA RNA HyperPrep kit incorporating short Y-adapters and barcoded PCR primers. The libraries were quantified with a fluorescent assay (dsDNA AccuGreen quantitation kit, Biotium) and checked for proper size distribution and average size with a TapeStation (D1000 Tape, Agilent). Library pools were then assembled and a 1× SPRI bead cleanup performed to remove traces of carryover PCR primers. The final library pool was quantified and run on an Illumina instrument (NextSeq, Novaseq).

### Data acquisition and preprocessing

**Figure 1.**
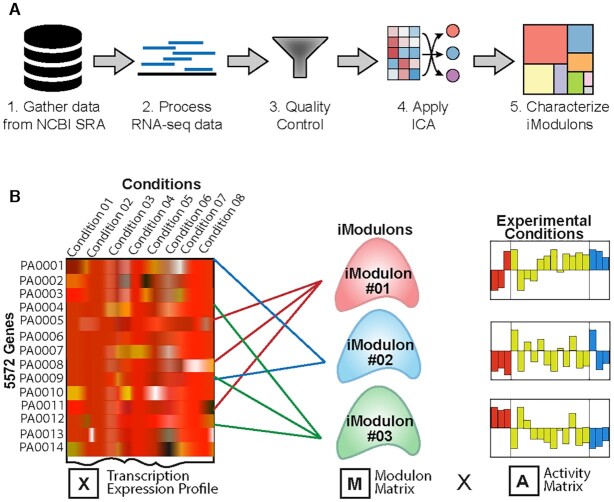
Data analysis procedure. (**A**) Overview of the methodology used in the study. It includes gathering high-quality data from the NCBI-SRA as well as generated in the lab. The RNAseq reads were processed and quality control was done. Further, the independent component analysis (ICA) was applied to generate the iModulons that were characterized to get the regulatory networks of *P. aeruginosa* (Adapted from Sastry *et al.* (7)). (**B**) ICA calculates the independently modulated sets of genes (iModulons). A compendium of expression profiles (X) is decomposed into two matrices: the independent components composed of a set of genes, represented as columns in the matrix **M**, and their condition-specific activities (A).

Apart from the in-house generated data, we also downloaded and processed all RNA sequencing data available from NCBI SRA for *P. aeruginosa* PAO1 (Figure 1A, B, and Supplementary Figure S2C). Data processing and quality control for the public datasets is detailed in Sastry *et al.* (7). Data processing and quality control scripts are available at https://github.com/avsastry/modulome-workflow. Briefly, raw FASTQ files were downloaded from NCBI using fasterq-dump (https://github.com/ncbi/sra-tools/wiki/HowTo:-fasterq-dump). Next, read trimming was performed using Trim Galore (https://www.bioinformatics.babraham.ac.uk/projects/trim_galore/) with the default options, followed by FastQC (http://www.bioinformatics.babraham.ac.uk/projects/fastqc/) on the trimmed reads. Next, reads were aligned to the *P. aeruginosa* genome (NC_002516.2) using Bowtie (31). The read direction was inferred using RSEQC (32) before generating read counts using featureCounts (33). Finally, all quality control metrics were compiled using MultiQC (34) and the final expression dataset is reported in units of log-transformed Transcripts per Million (log-TPM).

To ensure quality control, data that failed any of the following four FASTQC metrics were discarded: per base sequence quality, per sequence quality scores, per base n content, and adapter content. Samples that contained under 500 000 reads mapped to coding sequences were also discarded. Hierarchical clustering was used to identify samples that did not conform to a typical expression profile.

Manual metadata curation was performed on the data that passed the first four quality control steps. Information including the strain description, base media, carbon source, treatments, and temperature were pulled from the literature. Each project was assigned a short unique name, and each condition within a project was also assigned a unique name to identify biological and technical replicates. After curation, samples were discarded if (a) metadata was not available, (b) samples did not have replicates or (c) the Pearson R correlation between replicates was below 0.95. Finally, the log-TPM data within each project was centered to a project-specific reference condition. After quality control, the final compendium contained 364 high-quality expression profiles: 83 generated for this study, plus 281 expression profiles extracted from public databases (Supplementary Table S1).

### Computing robust independent components

To compute the optimal independent components, an extension of ICA was performed on the RNA-seq dataset as described in McConn *et al.* (35)

Briefly, the scikit-learn (*v*0.23.2) (36) implementation of FastICA (37) was executed 100 times with random seeds and a convergence tolerance of 10^–7^. The resulting independent components (ICs) were clustered using DBSCAN (38) to identify robust ICs, using an epsilon of 0.1 and minimum cluster seed size of 50. To account for identical components with opposite signs, the following distance metric was used for computing the distance matrix:


$$\begin{equation*}{{\rm{d}}_{{\rm{x}},{\rm{y}}}} = 1 - \left| {\left| {{\rho _{x,y}}} \right|} \right|\end{equation*}$$

where *ρ_x,y_* is the Pearson correlation between components *x* and *y*. The final robust ICs were defined as the centroids of the cluster.

Since the number of dimensions selected in ICA can alter the results, we applied the above procedure to the dataset multiple times, ranging the number of dimensions from 10 to 360 (i.e. the approximate size of the dataset) with a step size of 10. To identify the optimal dimensionality, we compared the number of ICs with single genes to the number of ICs that were correlated (Pearson *R* > 0.7) with the ICs in the largest dimension (called ‘final components’). We selected the number of dimensions where the number of non-single gene ICs was equal to the number of final components in that dimension.

### Determination of the gene coefficient threshold

The gene coefficients are determined as described in Sastry *et al.* (7). Each independent component contains the contributions of each gene to the statistically independent source of variation. Most of these values are near zero for a given component. In order to identify the most significant genes in each component, we iteratively removed genes with the largest absolute value and computed the D’Agostino *K*^2^ test statistic (39) for the resulting distribution. Once the test statistic dropped below a cutoff, we designated the removed genes as significant.

To identify this cutoff, we performed a sensitivity analysis on the concordance between significant genes in each component and known regulons. Known regulons were downloaded from RegPrecise (40). First, we isolated the 20 genes from each component with the highest absolute gene coefficients. We then compared each gene set against all known regulons using the two-sided Fisher's exact test (FDR < 10^–5^). For each component with at least one significant enrichment, we selected the regulator with the lowest p-value.

Next, we varied the D’Agostino *K*^2^ test statistic from 50 through 2000 in increments of 50, and computed the F1-score (harmonic average between precision and recall) between each component and its linked regulator. The maximum value of the average F1-score across the components with linked regulators occurred at a test statistic of cutoff of 420 for the *P. aeruginosa* dataset.

For future datasets where a draft TRN is unavailable, an alternative method is proposed that is agnostic to regulator enrichments. The Sci-kit learn (36) implementation of *K*-means clustering, using three clusters, can be applied to the absolute values of the gene weights in each independent component. All genes in the top two clusters are deemed significant genes in the iModulon.

### Regulator enrichment

The regulator enrichments are determined as described in Sastry *et al.* (7). The gene annotation pipeline can be found at https://github.com/SBRG/pymodulon/blob/master/docs/tutorials/creating_the_gene_table.ipynb. Gene annotations were pulled from Pseudomonas genomedb (41). Additionally, KEGG (42) and Cluster of Orthologous Groups (COG) information were obtained using EggNOG mapper (43). Uniprot IDs were obtained using the Uniprot ID mapper (44), and operon information was obtained from Biocyc (45). Gene ontology (GO) annotations were obtained from AmiGO2 (46). The known TRN was obtained from RegPrecise (40) and manually curated from literature. The performance of the predicted iModulons was evaluated using the ‘iModulon recall’ and ‘regulon recall’ values. The ‘iModulon recall’ represents the fraction of shared genes and the genes in an iModulon while ‘regulon recall’ is the fraction of shared genes and the genes in a regulon (Supplementary Figure S2B).

### Differential activation analysis

The distribution of differences in iModulon activities were determined as described in Rychel *et al.* (12). We fit a log-normal distribution to the differences in iModulon activities between biological replicates for each iModulon. For a single comparison, we computed the absolute value of the difference in the mean iModulon activity level and compared it against the iModulon's log-normal distribution to determine a *P*-value. We performed this comparison (two-tailed) for a given pair of conditions across all iModulons at once and designated significance as FDR < 0.01. Only iModulons with change in activity levels >5 were considered significant.

### Characterizing functionally correlated iModulons

The clustering iModulon activity is determined as described in Sastry *et al.* (7). Global iModulon activity clustering was performed using the clustermap function in the Python Seaborn (47) package using the following distance metric:


$$\begin{equation*}{{\rm{d}}_{{\rm{x}},{\rm{y}}}} = 1 - \left| {\left| {{\rho _{x,y}}} \right|} \right|\end{equation*}$$

where ||*ρ_x,y_*|| is the absolute value of the Pearson correlation between two iModulon activity profiles. The threshold for optimal clustering was determined by testing different distance thresholds to locate the maximum silhouette score.

### Prediction of the biosynthetic gene clusters

We used the antiSMASH algorithm to predict the BGCs in *P. aeruginosa* (30). While using the anti-SMASH software, we used the *P. aeruginosa* reference genome NC_002516.2 with the ‘relaxed’ detection strictness. The antiSMASH algorithm predicts different types of the BGCs like non-ribosomal peptide synthetases (NRPS), polyketide synthases (PKS), ribosomally synthesized and post-translationally modified peptides (RiPP), redox-cofactors and many more. Apart from the predicted BGCs, antiSMASH also provides the gene ontology annotations for the BGCs components.

### Generating iModulonDB Dashboards

iModulonDB dashboards were generated using the PyModulon package (7,16). Where applicable, we provide links to gene information in Pseudomonas.com (41).

## Results

### The iModulon structure of *Pseudomonas aeruginosa’*s transcriptome

We assembled the largest possible set of RNAseq profiles for *P. aeruginosa* from the literature and publicly available databases, and supplemented it with lab-generated RNAseq profiles for specific conditions of interest. We included RNA-seq expression profiles from two strains of *P. aeruginosa*, PAO1 and PAO1(*ΔmexB*). The PAO1(*ΔmexB*) was utilized in this study to potentially uncover transcriptional differences in stress response in a sensitized strain. The genomic and transcriptomic comparison analysis suggests that combining the data from both strains will not create a problem in ICA (Supplementary Note 2, Supplementary Figure S2A). The dataset included a range of growth conditions, including micronutrient supplementation, nutrition source variation, osmotic stress, iron starvation, and gene knockouts (Supplementary Figure S1).

**Figure 2.**
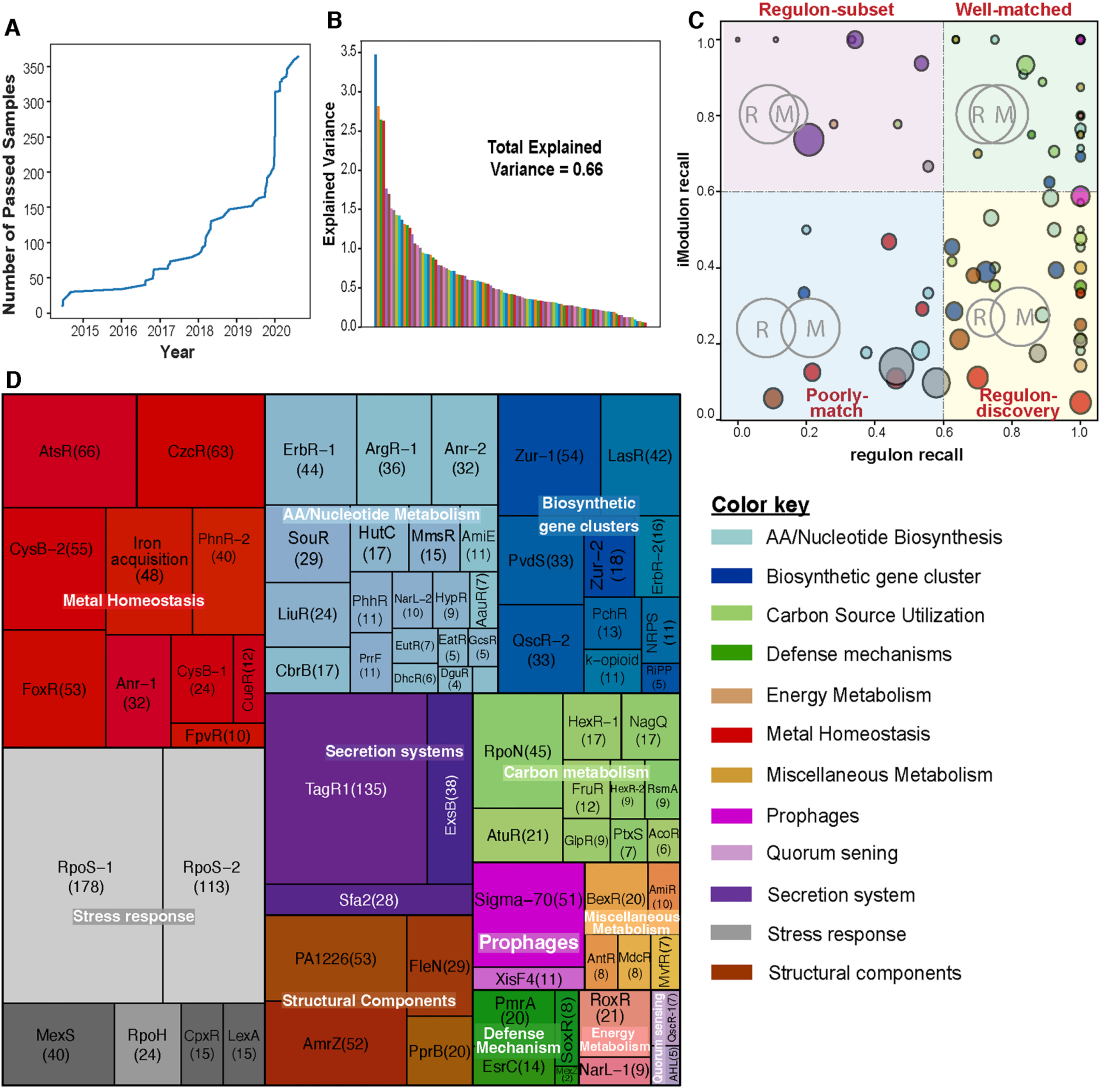
iModulons computed from the *Pseudomonas aeruginosa* transcriptomic data compendium. (**A**) Plot showing the amount of passed samples per year which is used in the study. (**B**) Bar plot showing the explained variance in all the iModulons with overall explained variance of 0.66. Total Explained Variance is the sum of the fraction of explained variance across all iModulons. (**C**) Scatter plot showing the regulon recall versus iModulon recall for all 104 iModulons found in the *P. aeruginosa* dataset. The scatter plot is divided into four quadrants: Upper right represents the well-matched iModulons; upper left shows iModulons representing a regulon-subset; lower right depicts the regulon-discovery; lower left contains the poorly-matched iModulons. The size of the circle represents the size of the iModulons (number of genes) and the color represents the functional categories as shown in the color key. (**D**) Treemap of the 104 *P. aeruginosa* iModulons. The size of each box represents the size of the iModulons (number of genes) and the color shades of each functional category represented by the explained variance of each iModulon. iModulons are grouped into 12 different categories: AA/Nucleotide Metabolism, Biosynthetic Gene Clusters, Carbon Metabolism, Defense Mechanism, Energy Metabolism, Metal Homeostasis, Miscellaneous Metabolism, Prophages, Quorum sensing, Secretion systems, Stress Responses and Structural Components. Abbreviations: AA, amino acids.

After filtering the profiles based on quality control criteria (see Materials and Methods), we compiled a transcriptomic compendium containing 364 samples (83 new +281 public expression profiles) named aeruPRECISE364 (Figure 2A and Supplementary Figure S1). All samples used for analysis were shown to have Pearson's correlation coefficient (PCC) of 0.97 between replicates, and to minimize possible batch effects, each individual experiment was normalized to a reference condition prior to calculating the iModulons (7).

iModulons represent a data-driven, top-down reconstruction of transcriptional regulatory networks and can be characterized using transcriptional regulator binding (11). To assign transcriptional regulators to iModulons, we compared each iModulon against regulons published in the literature. We compiled a TRN scaffold using RegPrecise (40), a manually curated database containing 58 regulons, and manually searched the literature for additional high-quality transcription factor binding sites. In total, the resulting TRN scaffold contained binding information for 134 TFs and corresponding regulons. This data is available in Supplementary Table S2.

We applied ICA to the transcriptomic compendium to identify independent signals in the data set that represent the effects of transcriptional regulators, resulting in the identification of 104 iModulons that explain 66% of the variance in the gene expression (Figure 2B). To annotate each iModulon, their genes were compared with those in the 134 regulons (Supplementary Table S3) to find statistically significant enrichments (see Materials and Methods). For iModulons with strong associations to known regulons, we used ‘iModulon recall’ and ‘regulon recall’ to evaluate our confidence in the associations. ‘iModulon recall’ represents the fraction of shared genes and the genes in an iModulon while ‘regulon recall’ is the fraction of shared genes and the genes in a regulon (Supplementary Figure S2B).

The relationship between the 134 regulons and the 104 iModulons are grouped into four categories (Figure 2C): well-matched, regulon subset, regulon discovery, and poorly matched. The well-matched category includes iModulons with a large fraction of shared genes with a known regulon, representing good agreement between our decomposition and the literature. The regulon subset category includes iModulons which capture a relatively small fraction of a known regulon, usually because the regulon is very large and iModulons only capture the most strongly regulated genes (or because of additional, unknown regulation). The regulon discovery category includes iModulons that contain most of a known regulon, but also include many other, typically uncharacterized genes. The poorly-matched category includes iModulons that are statistically significantly enriched for a known regulon, but their overlaps with the known regulons do not reach the threshold; they often correspond to master regulators, contain many uncharacterized genes, or are co-stimulated by several underlying signals. Thus, we identified 104 iModulons that are the regulated gene sets from complementary bottom-up and top-down methods.

### Functional classification of the iModulons, their coverage of genes, and how they form the variation in the RNAseq compendium

The 104 iModulons identified were annotated with different functions, such as BGCs, secretion systems, stress responses, prophages, metal homeostasis, structural components, amino acid metabolism, and carbon metabolism. We identified 11 iModulons related to BGCs, 14 related to metal homeostasis and 3 representing type III and type VI secretion systems (T3SS and T6SS, respectively) (48). We also functionally annotated iModulons associated with carbon, amino acids, sulfur, iron, secondary, lipid, and nitrogen metabolism (Figure 2D). Out of 104 iModulons, we found 22 that contain either uncharacterized genes, contain a single gene, and/or contain only genes with hypothetical function (Supplementary Table S3).

Among the 104 iModulons, four contain single genes. The remaining 100 iModulons contain 1835 unique genes. 561 genes were found in more than one iModulon. We have provided the information for each iModulon in the form of an interactive dashboard on iModulonDB.org (16). The dashboard is a user-friendly way for researchers to search for or browse the details of iModulons, TRN, genes, or regulators of interest. Such an examination gives both a guide to the study of molecular level mechanisms (49,50) and systems level mechanisms, such as those of resource allocation through changes in the transcriptome composition between conditions (11,15).

### iModulons provide a definition of genomic boundaries of biosynthetic gene clusters

**Figure 3.**
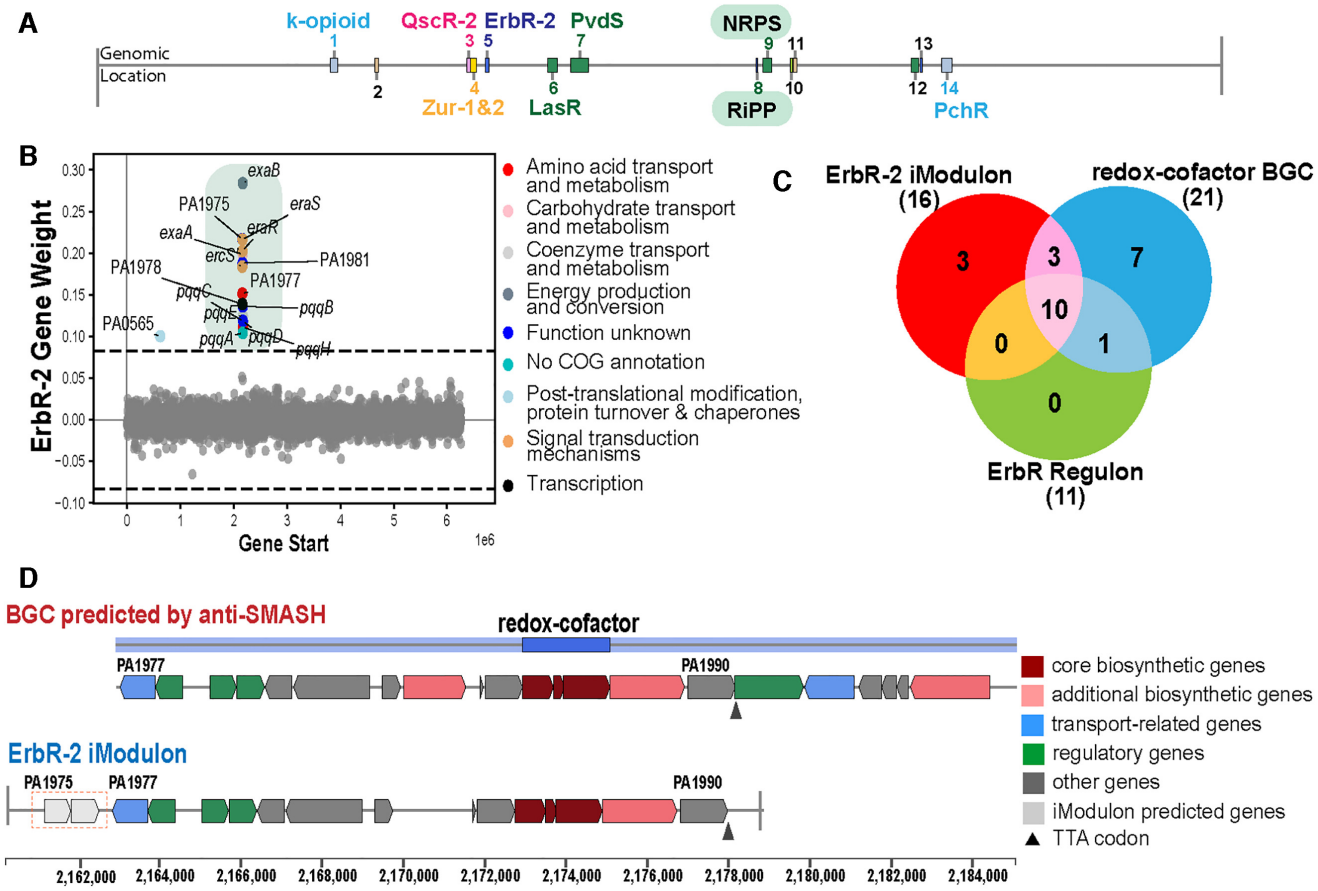
iModulons can aid in the definition of genomic boundaries of biosynthetic gene clusters (BGCs). (**A**) Genomic locations of the 14 predicted BGCs in the *P. aeruginosa* PAO1 by using the anti-SMASH software. (**B**) Scatter Plot showing the gene weights of the ErbR-2 iModulon with the color depicting the COG categories of the genes that it contains. (**C**) Venn diagram depicting the status of the genes in the ErbR-2 iModulons, ErbR regulon, and the predicted redox-cofactor BGCs by using the anti-SMASH software. (**D**) Genomic overview of the redox-cofactor BGCs predicted by the anti-SMASH software, alongside the iModulons whose boundaries are defined by genes between the PA1975-PA1990.

The 104 iModulons identified contain 11 out of the 14 predicted BGCs in *P. aeruginosa* using anti-SMASH software (Figure 3A and Supplementary Figure S3A). The remaining 3 BGCs had fairly normally distributed transcriptional activity in the conditions represented in the dataset analyzed, and thus were not detected by ICA. The ErbR-2 iModulon contains coregulated genes which are predicted to be redox-cofactors, such as pyrroloquinoline quinone (PQQ) (Figure 3B and C). The BGC’s boundaries defined by antiSMASH are arbitrarily marked from PA1977–PA1997. However, the iModulon captured by ICA identifies an independent transcriptional signal from PA1975 to PA1990 (Figure 3D). All 11 identified iModulons related to BGCs can be used to annotate their boundaries (Supplementary Figure S3B).

### iModulons elucidate responses to N-acetylglucosamine as the sole carbon source

Chronic infections with *P. aeruginosa* in CF patients can lead to increased lung deterioration and higher mortality rates (51). Previous research found that genes required for N-acetylglucosamine (GlcNAc) metabolism, such as the NagQ operon, were upregulated during *in vitro* growth in sputum from CF patients (52,53). It is hypothesized that *P. aeruginosa* is able to take up GlcNAc from various sources, such as host mucin or bacterial peptidoglycan, during infection (52,54). Therefore, the role of GlcNAc in driving *P. aeruginosa* virulence and persistence provided an impetus for additional data generation and analysis.

**Figure 4.**
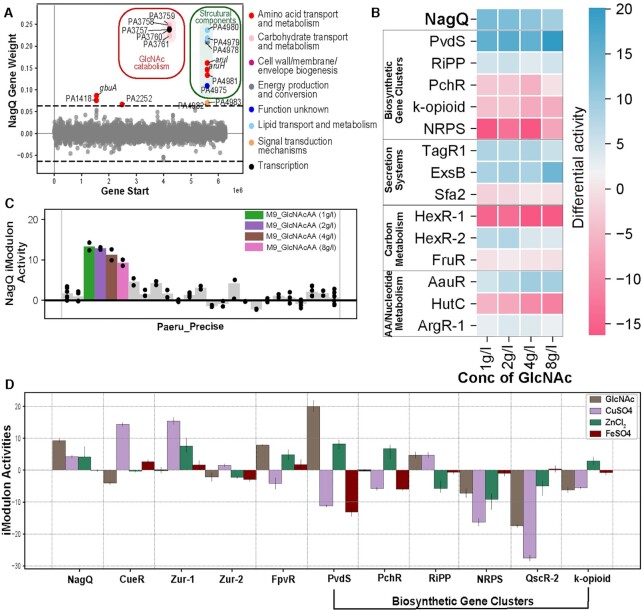
iModulon responses to GlcNAc culture. (**A**) Scatter Plot showing the gene weights of the NagQ iModulon; the color depicts the COG categories. The NagQ iModulons have two regulons; one is GlcNAc catabolism and other is related to structural components. (**B**) Heat map depicting the activity of selected iModulons in different concentrations of GlcNAc (1g/l, 2g/l, 4g/l, and 8g/l). It describes the change in differential activities in NagQ, biosynthetic gene clusters, secretion systems, carbon metabolism, amino acid metabolism, and nucleotide metabolism. (**C**) Activity plot of the conditions expressed in NagQ iModulon in the Paeru_Precise. (**D**) Plot showing iModulon activities in the presence of *N*-acetyl glucosamine (GlcNAc), ZnCl, CuSO_4_ and FeSO_4_ micronutrients. The iModulons include the micronutrient metabolism (NagQ, CueR, Zur-1, Zur-2, FpvR) and the biosynthetic gene clusters (PvdS, PchR, RiPP, NRPS, QscR-2 and k-opioid).

We grew *P. aeruginosa* PAO1 in M9 minimal media supplemented with different concentrations of GlcNAc (1, 2, 4 and 8 g/l) as the sole carbon source and examined its impact on the identified iModulons (Figure 4A-C). Among the BGC iModulons, we found an increased expression of the PvdS iModulon, which includes the genes involved in the regulation and synthesis of the siderophore pyoverdine (51). The increased expression of siderophores in the presence of GlcNAc has been previously reported in the *Streptomyces* species (55,56) but not in *P. aeruginosa*. In contrast to the increased expression of the PvdS iModulon, we found decreased expression of the QscR-2 iModulon, which contains a pyocyanin-associated BGC during growth in GlcNAc-M9 media.

Our analysis also found a previously unannotated, ribosomally synthesized and post-translationally modified peptide (RiPP) BGC iMoudlon, which showed increased expression during bacterial growth on GlcNAc as the sole carbon source. The novel RiPP iModulon contains genes encoding DUF692-associated bacteriocin, as predicted by anti-SMASH (Supplementary Figure S4A). This DUF692-associated bacteriocin has not been reported in *P. aeruginosa*, however the evidence of its presence is reported in *Streptomyces* and *Methanobacteria* sps (57). The BGC RiPP iModulon was also found to be expressed in the presence of sodium hypochlorite (NaOCl) (Supplementary Figure S4B). Hypochlorite is known to be elevated in CF sputum (58,59) as well as facilitate bacterial clearance by infiltrating neutrophils in the lung, although a previous study showed that the production of hypochlorite was diminished in the phagosomes of neutrophils from CF patients (60). Bacteriocins are bacterially-derived antimicrobial peptides secreted as part of interbacterial competition (61). The upregulation of this BGC iModulon under these experimental conditions suggests its role in helping *P. aeruginosa* defend against competitors during decreased nutrient availability or stress.

### iModulons highlighted bacterial response to metal micronutrient supplementation

Some studies report that the sputum sample of CF fibrosis patients shows an elevated concentration of various metals like Ca, Mg, Mn, Zn, Mo and Ni (62,63). In our study, we used the PAO1 and PAO1(*ΔmexB*) strains to generate the RNAseq profile data in different micronutrient conditions and checked their expression, as many previous papers suggest that micronutrient concentrations are important factors in the pathogenicity and virulence of *P. aeruginosa*. We generated the transcriptomic profiles for Cu, Zn, and Fe, and examined the iModulon activities in their presence (Figure 4D), which were included in the RNAseq dataset used for ICA. We found that the CueR iModulon is upregulated in the presence of Cu, as expected. The expression of the Zur-2 and FpvR iModulons are repressed in the presence of Zn and Fe, respectively. Both Zur-2 and FpvR function in concert with other proteins to bring in Zn and Fe, respectively, into bacteria during growth in conditions with low Zn or Fe (64,65). The Zur-1 iModulon shows activation in the presence of Zn because the genes responsible for binding the Zn has negative gene weight. Interestingly, we also found that iModulons related to the secretion of pyochelin and pyoverdine, as well as a novel bacteriocin producing (RiPP), are upregulated in the presence of these micronutrients. The PchR and PvdS iModulons are responsible for the expression and secretion of the siderophores pyochelin and pyoverdine, respectively. The PchR iModulon showed increased activity in the presence of Zn, while PvdS is activated during growth with both GlcNAc and Zn. Both pyochelin and pyoverdine are known to be important in *P. aeruginosa* pathogenicity (66), which further supports that the novel RiPP BGC, which has similar expression profiles, may also play a role in the ability of the pathogen to infect or invade host lungs.

### iModulons reveal coordinated expression of secretion systems

Secretion systems play a role in the pathogenicity of *Pseudomonas* by facilitating the secretion of virulence factors (67). We found increased expression of the H1-T6SS and T3SS secretion systems in the TagR1 and ExsB iModulons respectively during growth on GlcNAc as the sole carbon source (Figure 4B). H1-T6SS is known to target other prokaryotes and contributes to the survival advantage of *P. aeruginosa* (68). In comparison, the T3SS secretion system in *P. aeruginosa* is a major virulence factor that contributes to cytotoxicity and acute infections. T3SS is used to inject the effector proteins into the host cells (69). We hypothesize that the activation of the secretion systems during growth on GlcNAc might be helpful to export the products of the BGCs, such as RiPP, outside *P. aeruginosa*, furthering their fitness advantage in limited nutrient conditions (69).

### iModulons describe central metabolic pathways

**Figure 5.**
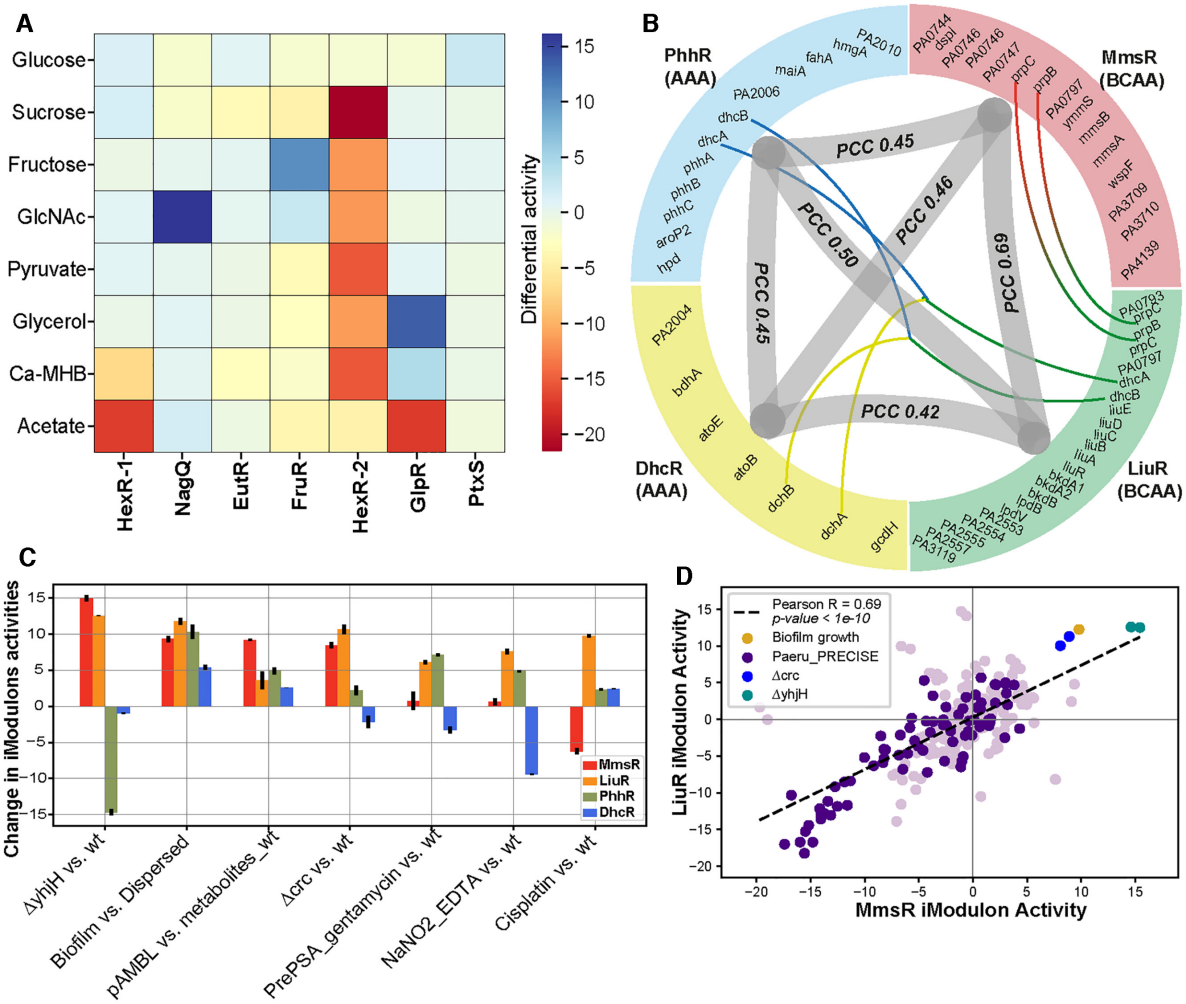
iModulons related to Carbon metabolism and Amino acid/Nucleotide metabolism. (**A**) Heat map depicting the differential activity of glucose, sucrose, fructose, N-acetylglucosamine, pyruvate, glycerol, Ca-MHB (bacteriological media), and acetate with respect to HexR-1, NagQ, EutR, FruR, HexR-2, GlpR, and PtxR iModulons. (**B**) Correlation plot among the Branched chain amino acid [BCAA (LiuR and MmsR)] and the Aromatic amino acid [AAA (DhcR and PhhR)]. The outer layer is divided into the four arcs which depict the four different iModulons. Thin lines represent the common genes among the iModulons, and the thick line connecting different iModulons depicts the Pearson correlation coefficients (PCC). (**C**) Bar plot representing the iModulon activities of MmsR, LiuR and PhhR under different conditions. The x-label shows some conditions used in the study. The ‘△yhjH vs. wt’ is the knockout of the yhjH, ‘Biofilm vs. Dispersed’ is the biofilm mode of growth, ‘pAMBL vs. metabolite_wt’ is the pAMBL plasmid showing overexpression of metabolites, ‘△crc vs. wt’ is the deletion of the global regulator of crc, ‘PrePSA_gentamycin vs. wt’ is the pre-PatH-Cap library of *P. aeruginosa* (‘PSA’ PAO1-GFP) treated with gentamycin,’NaNO2_EDTA vs. wt’ is the presence of sodium nitrite and EDTA in the media, and ‘Cisplatin vs. wt’ is the presence of cisplatin and bile in the media. (**D**) Scatter plot showing the correlation between the BCAA pathways iModulons, i.e. LiuR and MmsR, with the PCC of 0.69.

We found multiple iModulons related to central carbon metabolism (Supplementary Figure S5). Among the identified metabolic iModulons, NagQ is involved in the catabolism of GlcNAc to fructose-6-phosphate, a key glycolytic intermediate. As mentioned previously, GlcNAc has been suggested to play a role in the pathogenicity of *P. aeruginosa* in CF patients (52). Furthermore, iModulons related to the catabolism of the ethanolamine, glycerol, fructose, and 2-ketogluconate describe the state of the metabolic network when these substrates serve as the sole carbon source in place of the preferred glucose (Figure 5A, Supplementary Figures S5 and S6A). This demonstrates the ability of iModulons to highlight the complex metabolic network of *P. aeruginosa* that contributes to its ability to grow in diverse environments (70,71).

Several of the identified iModulons mapped onto amino acid metabolic pathways, such as branched chain amino acids (MmsR, AtuR, PrrF, and LiuR), aromatic amino acids (PhhR and DhcR), arginine catabolism (CbrB), histidine utilization (HutC), arginine succinyltransferase (ArgR-1 & 2), arginine deaminase (ArcR), and L-hydroxyproline (HypR) (Supplementary Figure S5). We found significant correlations among the iModulons regulating the branched-chain amino acid (BCAA) and aromatic amino acid (AAA) pathways (Figure 5B). It is known that amino acids are the main nutrient source for *P. aeruginosa* in CF lungs (53,71) and it is hypothesized that they play a vital role in promoting biofilm formation (72) in CF patients (73). Our data showed that iModulons related to amino acid metabolism pathways had higher activities during growth in biofilm conditions compared to planktonic growth (Figure 5C and Supplementary Figure S6B). This is important as *P. aeruginosa* primarily grows as a biofilm in CF lungs (74), and a microarray study looking at gene expression at different timepoints of *P. aeruginosa* infections in CF patients showed increased expression of amino acid metabolism genes (71).

### iModulons related to the altered metabolism of branched-chain amino acids

Bis-(3′-5′)-cyclic dimeric guanosine monophosphate (c-di-GMP) is a secondary messenger that regulates various important cellular processes like quorum sensing, biofilm formation, and pathogenicity (75). YhjH is a c-di-GMP phosphodiesterase and, upon induction, it decreases c-di-GMP levels (76). A decrease in c-di-GMP levels leads to a decrease in the biofilm formation and increased biofilm dispersal. We found the knockouts of YhjH (△yhjH, PRJNA381683) (77) led to increased expression of BCAA metabolism iModulons, subsequently increasing intermediates of the tricarboxylic acid (TCA) cycle (Figure 5C). Likewise, the deletion of Crc (△*crc*) also led to increased expression of the BCAA iModulons (Figure 5D), similar to YhjH. Crc is a global metabolic regulator that represses succinate metabolism and BCAA assimilation in *P. aeruginosa* and *P. putida* (78). Therefore, from our analysis, we can hypothesize that YhjH and Crc may be used as an important target to control the biofilm formation and pathogenicity of *P. aeruginosa* through the alteration of BCAA metabolism. Activities of the identified iModulons were therefore able to untangle complex relationships between metabolites, transcriptional regulators, and lifestyle in *P. aeruginosa*.

### Correlated activity changes of iModulons lead to the definition of Stimulons

**Figure 6.**
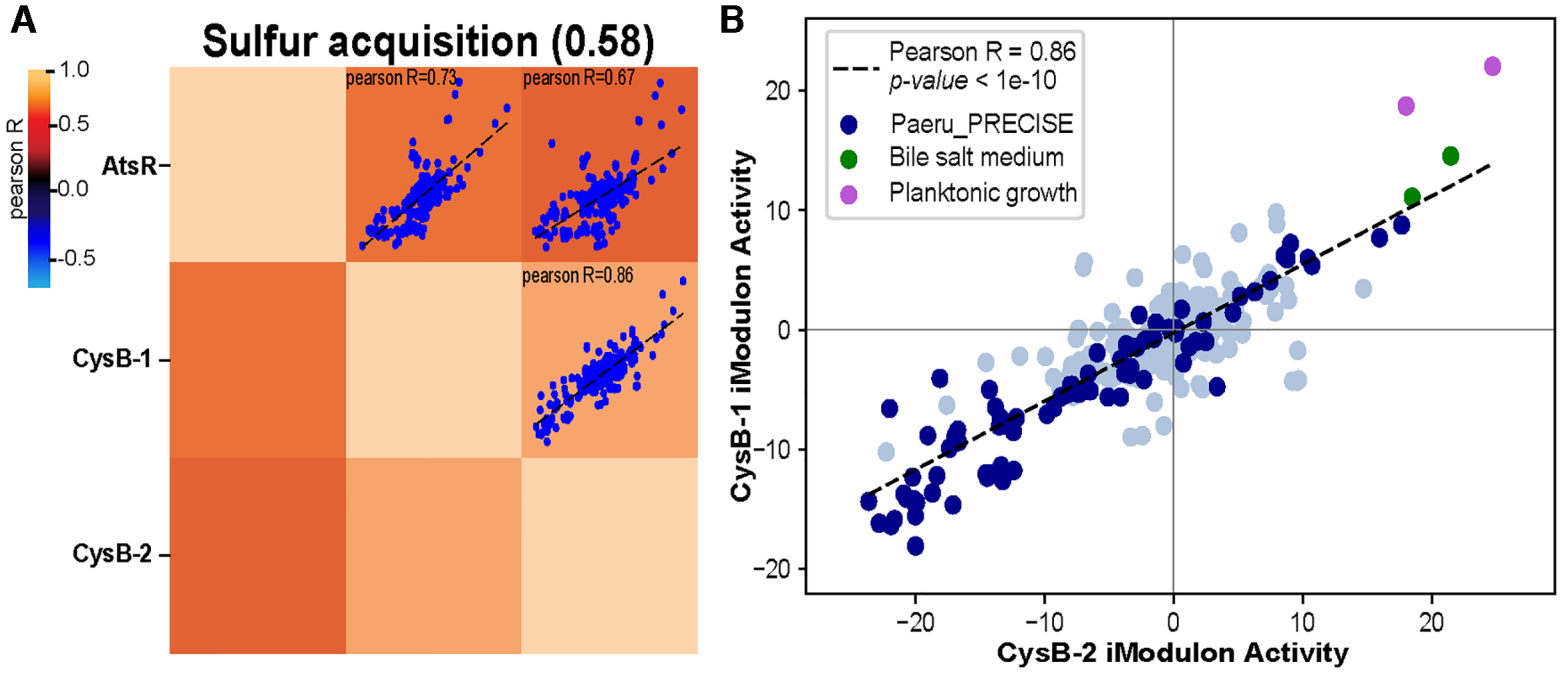
Activity clustering of the iModulons among *P. aeruginosa* defines stimulons. (**A**) Sulfur acquisition cluster includes the grouping of AtsR, CysB-1 and CysB-2 iModulons with silhouette score of 58. (**B**) The scatter plot shows correlation between the CysB-1 and CysB-2 iModulons with PCC of 0.86. Both the iModulons show high activity in the planktonic condition and bile salt medium of *P. aeruginosa*.

We have clustered the iModulons based on their correlation as a set (Figure 6A and Supplementary Figure S7B). Though iModulons are independently modulated throughout the transcriptome, clusters of iModulons may be similarly expressed across most conditions in the compendium and only diverge from one another under select conditions. Thus, a cluster of iModulons with coordinated activity changes can be interpreted as a ‘stimulon’. Such clusters of iModulons are of interest for understanding the broader structure of transcriptional regulation (79). For example, we have found that sulfur stimulon {AtsR, CysB-1 and CysB-2} and iron stimulon {FpvR, PvdS, PchR, FoxR and Iron acquisition} are among the top clustered stimulons in *P. aeruginosa*.

*Sulfur acquisition:* The iModulons AtsR, CysB-1 and CysB-2 form a sulfur acquisition stimulon (Figure 6A). AtsR is a transcription factor that encodes the ABC transporters of sulfate and other ions. We observed that the AtsR iModulon was activated during oxidative stress (paraquat treatment) (Supplementary Figure S7A). The relationship between sulfate limitation and the oxidative stress response has been previously established in *E. coli* (80) but not in *P. aeruginosa*. The CysB-1 and CysB-2 regulators modulate sulfur uptake and cysteine biosynthesis, as well as influence the genes involved in host colonization and virulence factor production (81). The two CysB iModulons are highly expressed in planktonic growth conditions as well as in the presence of bile (Figure 6B). However, CysB’s direct connection with bile has not been previously established in the literature. Taurine, a sulfur-containing amino acid, is one the primary components of bile acids. We hypothesize that *P. aeruginosa* upregulates its sulfur acquisition genes in response to the presence of taurine in the conjugated bile acid. Interestingly, in certain patients with CF, there can be microaspirations of bile into the lungs, and studies have shown bile to affect the transition of *P. aeruginosa* into biofilms (82). Therefore, it is possible that CysB may play an important role in the pathogenicity of *P. aeruginosa* in CF lungs through its role in acquiring sulfur from bile aspirations.

*Iron acquisition:* We identified a stimulon of five iron-related iModulons (FpvR, PvdS, PchR, FoxR and Iron acquisition) (Supplementary Figure S7B). The five iModulons involved in this cluster contained genes involved in the uptake of iron through endogenous (pyoverdine, *fpv, pvd*) or exogenous (xenosiderophores, FoxR and heme) carriers (83). The activities of both the endogenous PvdS and exogenous FoxR iModulons were upregulated during the presence of the chelator EDTA and during planktonic growth (PCC 0.67) (Supplementary Figure S7C). The iron acquisition iModulon was previously uncharacterized and known as Uncharacterized-13, which was further annotated to be involved in iron acquisition by clustering analysis. Additionally, the presence of an uncharacterized iModulon (Uncharacterized-13) in this cluster allowed us to annotate its potential function, which we hypothesize as playing a role in pyoverdine synthesis. Several genes, such as PA2531, PA4709, *phuR*, *opmQ*, *pvdT*, *pvdR*, *pvdE* and PA2412 are shared between the PvdS and Uncharacterized-13 iModulons (Supplementary Figure S7D). Thus, our analysis provides insight into the interconnectedness of iron acquisition systems in *P. aeruginosa*.

### iModulons show ‘Fear vs. Greed’ Trade-off

In previous studies of *E. coli* and *S. aureus* transcriptional regulation, a trade-off between the expression of translation machinery and stress-hedging genes was observed (10,11). This global trade-off was termed the ‘Fear vs. Greed’ trade-off.

**Figure 7.**
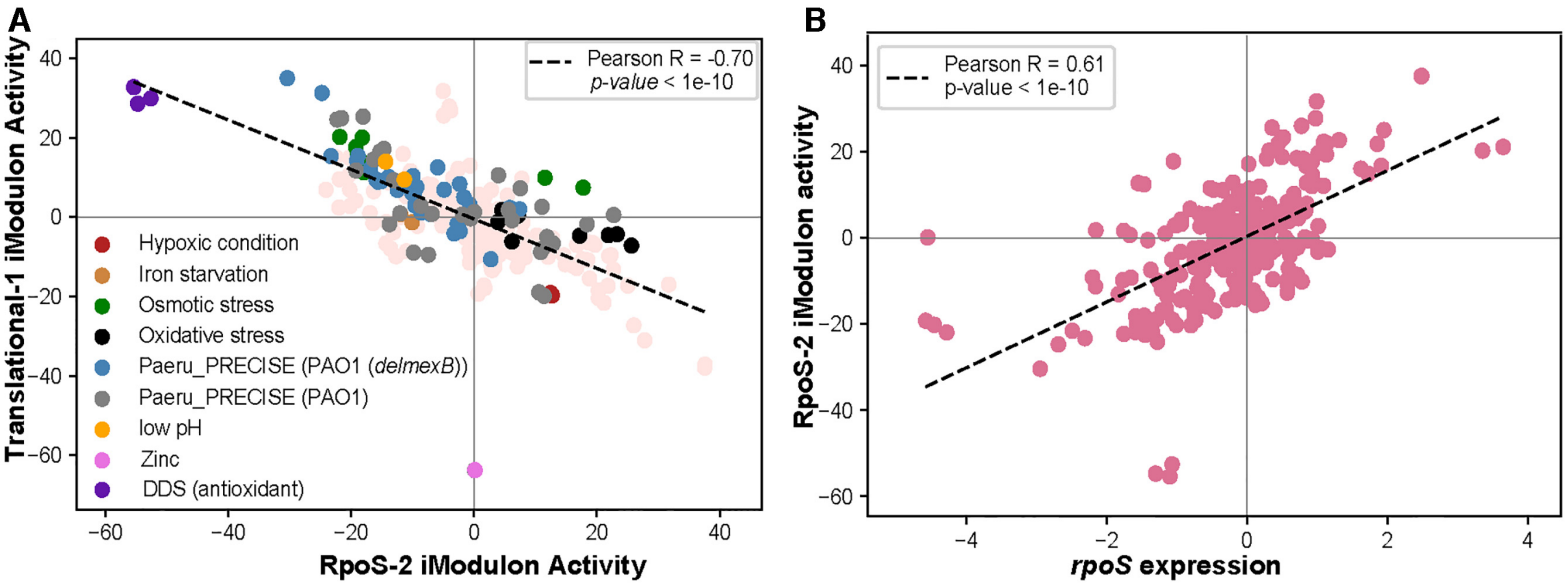
Fear versus Greed trade-off relationship between iModulons. (**A**) The RpoS-2 iModulon activities were anti-correlated with the Translational-1 iModulon activities. All the stress conditions (hypoxia, iron starvation, osmotic stress, oxidative stress, and low pH) were highlighted with different colors. (**B**) Scatter plot showing correlation between the RpoS-2 iModulon activity and the rpoS gene expression with the Pearson's correlation coefficient of 0.61.

The allocation of the resources to the optimal growth (greed) versus its allocation towards the bet-hedging strategies to attenuate its effect of the stressors in the environment (fear) (11,84) was demonstrated using two iModulons in *E. coli*. We identified two iModulons in *P. aeruginosa* (Translation-1 and RpoS-2 iModulons) that were orthologous to these *E. coli* iModulons (translation and RpoS iModulons) (Supplementary Table S4). The RpoS-2 iModulon includes the sigma factor RpoS, which is a central regulator of the bacterial response to stress that allows cells to survive environmental challenges. The translational iModulon represents the translational machinery like ribosomal proteins and the growth-related function of the transcriptome. We identified an anti-correlation relationship between the RpoS-2 iModulon and the Translational-1 iModulon (Figure 7A). Further, the RpoS-2 iModulon also showed correlation (PCC 0.61, *P*-value < 10^–10^) with the expression level of the *rpoS* gene. High correlation between the activity of the RpoS iModulon and *rpoS* gene expression was also observed in *E. coli* (Figure 7B). These results suggest that the ‘Fear vs Greed’ trade-off relationship is conserved among bacterial species.

## DISCUSSION

We have constructed a large compendium of *P. aeruginosa* transcriptomes from all publicly available high-quality data, compiled a TRN of 134 regulons from literature, and computed and characterized a data-driven TRN of 104 iModulons that matches well with the literature. The regulons are based on targeted biomolecular studies, whereas iModulons result from the data analysis of a global compendium of transcriptomic data. These complementary approaches to TRN elucidation synergize well. The iModulons were effective for clarifying BGC groups (including identifying a new BGC), characterizing simple and disease-relevant growth conditions from a transcriptomic perspective, clustering functional groups of genes and comparing regulatory modules across organisms.

From our analysis, we find that iModulons are useful in quickly determining the boundary of BGCs without the need to generate specific gene knockouts or heterologous expression strains. Various initiatives have been undertaken to confirm the boundaries of BGCs, but existing, arbitrary rules do not capture the important feature that they be co-transcribed (85–87). Thus, in this study, we present improved annotations for 11 *P. aeruginosa* BGCs. Interestingly, we identified several iModulons of BGCs and secretion systems that could play a significant role in establishing *P. aeruginosa* infections in the lungs of CF patients. Several BGCs, including the newly discovered RiPP discussed earlier, are upregulated under the GlcNAc and supplemented Cu growth environments. Both GlcNAc and Cu have been previously shown to be altered in the microenvironments of the CF lungs (52,62). We also identified upregulation of iModulons related to the different secretion systems (H1-T6SS and T3SS) in the presence of the GlcNAc. These secretion systems are well known to increase the pathogenicity in the host (69,88). Thus, we hypothesize that the BGCs and the secretion system might play an important role in pathogenesis of *P. aeruginosa*. Furthermore, Bernier *et al.* demonstrated that several amino acids, at concentrations found in CF sputum, promoted biofilm formation of *P. aeruginosa* through the alteration of c-di-GMP signaling (73). In our study, we found that the AAA and the BCAA related iModulons were upregulated in the biofilm mode of growth. This highlights the ability of iModulons to identify important physiological changes that impact *P. aeruginosa* metabolism and fitness in altered environmental conditions, such as in the lungs of patients with CF.

From the functional clustering of iModulons, we annotated a previously uncharacterized iModulon (Uncharacterized-13) that may be involved in additional iron acquisition. Furthermore, we found a potential correlation of the bile and sulfur acquisition, which might be an important factor for *P. aeruginosa* infection in CF patients. We performed interspecies iModulon comparison using our in house python function available in Pymodulon package (7). We found 20 iModulons from *P. aeruginosa* showing high correlation with the *E. coli* iModulons (Supplementary Table S4), with the translational iModulon being the most important among them. Additionally, we find that the stress related iModulon (RpoS-2) shows anti-correlation with the translational (Translational-1) iModulon, which demonstrates the survival strategy of *P. aeruginosa* under stress conditions in a ‘Fear-vs-Greed’ trade-off modality.

All the activity and expression profiles as well as the details of iModulons would be very useful for microbiologists to understand the large transcriptional plasticity of *P. aeruginosa*. The code for this pipeline is available on Github. The framework of the Pseudo Precise iModulons would be helpful to elucidate the regulatory metabolic networks, transcription factors, and various cross-talk among mechanisms. To browse or search dashboards for each iModulon and gene analyzed in this study, visit iModulonDB.org (https://imodulondb.org/dataset.html?organism = p_aeruginosa&dataset = precise364).

Previously, two studies used the network based methods to identify the gene regulatory networks of *P. aeruginosa* (89,90). However our iModulon approach is distinct and more comprehensive than previous two approaches (Supplementary Table S5). In this study, we implemented machine learning to identify the TRN in *P. aeruginosa*. We incorporated high quality transcriptomics data, both in-house generated as well as all publicly available data from the SRA database, to get the independently co-regulated sets of genes (iModulons) which provide a genome-wide, top-down perspective of the TRN of *P. aeruginosa*. We have demonstrated its usefulness for characterizing BGCs, metabolism, and virulence, and its wide scope could enable additional insights into many other processes in *P. aeruginosa*. It may also serve as the basis for comparisons in regulation across the phylogenetic tree, as we have demonstrated with *E. coli*.

## DATA AVAILABILITY

All the in-house generated sequences were deposited in the NCBI-Sequence Read Archive database (PRJNA717794). The accession number of the deposited reads is provided in the Supplementary Table S1. While the X, M and A matrices are available on GitHub. Each gene and iModulon have interactive, searchable dashboards on iModulonDB.org, and data can also be downloaded from there.

## DATA AVAILABILITY

The customized code for the ICA analysis is provided on GitHub. As well as various files including the X, M, A matrices, TRN regulator file, gene annotated files, gene ontology and kegg pathway annotation files are available on GitHub (https://github.com/akanksha-r/modulome_paeru1.0).

## Supplementary Material

gkac187_Supplemental_Files
