# Supplementary material for: Machine learning from Pseudomonas aeruginosa transcriptomes identifies independently modulated sets of genes associated with known transcriptional regulators
Source: Nucleic Acids Res. 2022 Mar 31;50(7):3658–72. doi: 10.1093/nar/gkac187 (PMC9023270; doi:10.1093/nar/gkac187)
Supplement: gkac187_Supplemental_Files [file gkac187_supplemental_files.zip › R2_Suppl_info_NAR_aeruPRECISE364.pdf]

Machine Learning from *Pseudomonas aeruginosa* transcriptomes identifies independently modulated sets of genes associated with known transcriptional regulators

Akanksha Rajput[1], Hannah Tsunemoto[2], Anand V. Sastry[1], Richard Szubin[1], Kevin Rychel[1], Joseph Sugie[2], Joe Pogliano[2], Bernhard O. Palsson[1,3,4,5]*

Author Affiliations

[1]Department of Bioengineering, University of California, San Diego, La Jolla, USA
[2]Division of Biological Sciences, University of California San Diego, La Jolla, CA 92093, USA
[3]Department of Pediatrics, University of California, San Diego, La Jolla, CA, USA
[4]Center for Microbiome Innovation, University of California San Diego, La Jolla, CA 92093, USA
[5]Novo Nordisk Foundation Center for Biosustainability, Technical University of Denmark, Kemitorvet, Building 220, 2800 Kongens, Lyngby, Denmark

Correspondence
*To whom correspondence should be addressed:
Bernhard O. Palsson
University of California, San Diego
9500 Gilman Drive
La Jolla, CA 92093
palsson@ucsd.edu

Supplementary Notes

Supplementary Note 1. Evaluation of the batch effect
Supplementary Note 2. Difference between the PAO1 and PAO1 (ΔmexB) strains

Supplementary Figures

Supplementary Figure S1. Evaluation of the batch effect after the normalization. *The principal component plot shows the diversity of the samples used in the study. The color represents different projects used in the study. A) before normalization, b) after normalization. The clustermap shows the global correlations between one sample and all others along with the hierarchical clustering. C) before normalization, and D) after normalization.*

Supplementary Figure S2. Overview of the RNAseq data of Pseudomonas aeruginosa used in the study. *A) Differential activation analysis showing the difference in the activities (104 iModulons) of the samples in PAO1 and PAO1(ΔmexB). From the in-house generated samples, we plotted M9fructAA, M9GlcNAcAA, M9pyruvAA, M9sucAA, M9glucAA, and M9glucAA_NaCl to compare the iModulon activities in both the PAO1 and PAO1(ΔmexB). B) Schematic representation of the definitions of regulon recall and iModulon recall. C) Detailed overview of the methodology used in the study. It includes decomposition, outlier detection, characterization, and interpretation of the iModulons. (Adapted from Sastry et al. (1)).*

Supplementary Figure S3. Overview of the biosynthetic gene clusters (BGCs) of Pseudomonas aeruginosa predicted from our ICA based analysis. *A) Genomic location of the 14 BGCs predicted from the antiSMASH software. B) structure of 11 BGCs defined by our ICA based pipeline*

Supplementary Figure S4. Novel RiPP iModulon. *A) Scatter plot showing the gene weights of the novel RiPP iModulon, the color depicts the COG categories. B) Activity plot of the conditions expressed in RiPP iModulon in the Paeru_PRECISE*

Supplementary Figure S5. *Graphical representation of the iModulons predicted and mapped as carbon and amino acid metabolism pathways. The carbon metabolism pathways are ED pathway, glycolysis, and peripheral pathways. While the amino acid metabolism pathways includes the branched chain amino acid (BCAA), aromatic amino acid (AAA), histidine utilization (HUT), arginine decarboxylase (ADC), arginine succinyltransferase (AST), arginine deiminase (ADI), and L-hydroxyproline (LHP) pathways*

Supplementary Figure S6. iModulons related to the Carbon metabolism and the Amino Acid/Nucleotide metabolism. *A) Activity plot of the conditions expressed in GlpR iModulons in the Paeru_PRECISE; where M9glyAA is the samples collected in M9 media with glycerol and amino acids. B) Scatter plot showing the correlation between the BCAA pathways iModulons i.e.*

LiuR and PhhR with the PCC of 0.50; where M9sucAA is the samples collected in M9 media with sucrose and amino acids.

Supplementary Figure S7. Activity clustering of the iModulons among the P. aeruginosa. *A) Differential iModulon activity (DIMA) plot of the M9glucAA and Paraquat v/s the control condition showing the upregulation of the AtsR iModulon. B) Iron acquisition cluster with iModulons like FpvR, PvdS, Uncharacterized-13, PchR, and FoxR grouped with silhouette score of 0.51. C) The scatter plot shows the correlation between the FoxR and PvdS iModulons with PCC of 0.67. Both the iModulons show high activity in the EDTA and planktonic form of growth in P. aeruginosa. D) Scatter plot showing the gene weights between the Uncharacterized-13 and the PvdS iModulons. The red-colored genes are common between both iModulons.*

Supplementary Notes

Supplementary Note 1. Evaluation of the batch effect

To show that batch effects are dramatically decreased, we provide **Supplementary Figure S1** which includes PCA plots and clustermaps before and after normalization. Prior to normalization, the first PC is dominated by a single batch and the second PC by a single condition, with most samples falling near 0 for the first two PCs because the variance due to batch effects dwarfs biological effects. After normalization, the top two PCs spread conditions much more evenly, which is consistent with biological variation playing a more dominant role. We also have clustermaps which show a large increase in correlation between samples and more meaningful associations after normalization. It is difficult to perform an actual statistical test for batch effects here because we do not have matched conditions from separate batches, so any test would mix biological and batch-dependent variables.

Also, note that ICA seeks to find genes that are co-regulated as independent signals throughout the data. When we center each batch to a reference condition, we preserve only the relative changes in the remaining conditions, which should rely on the same underlying biology as all other batches. We acknowledge that the magnitude of the change in gene expression/iModulon activity could depend on the batch, and that is part of why we cannot and do not compare conditions from separate batches in our downstream analyses. Also, if a batch effect affected only a subset of genes, they would likely be picked up as their own iModulon and could easily be ignored when drawing biological conclusions from other iModulon activities – indeed, ICA has been used for batch correction in the past (2–4). These points combine with our multi-dataset *E. coli* study (5) to make us confident that the biological gene modules we discuss in this publication are not simply technical noise resulting from batch effects.

Supplementary Note 2. Difference between the PAO1 and PAO1 (ΔmexB) strains

Prior transcriptomic ICA decompositions have successfully combined closely related strains, such as MG1655 and BW 25113, or those which differ by gene knock-outs or other strain differences (6). To quantify how different these two strains are, we compared their reference genomes using BLAST (blastn) (7). The genome comparison analysis suggests that both the strains are highly similar with a max score, total score, query cover, evalue and %identity of 1.069e+07, 1.231e+07, 100%, 0.0, and 100%, respectively. The high similarity suggests that combining RNAseq data from both strains will not create a problem with the ICA decomposition of the data set (8).

ICA can be used to find the transcriptomic difference between strains. Since we had matched conditions for both strains (M9fructAA, M9GlcNAcAA, M9glucAA_NaCl, M9glucAA, M9pyruvAA, and M9sucAA), we compared the activity levels for each individual pair and in aggregate. The aggregate differential iModulon activity plot (DIMA) is shown in **Supplementary Figure S2A**. To statistically compare the iModulons, we fit a log-normal distribution to the differences in iModulon activities between biological replicates for each iModulon. For a single comparison, we computed the absolute value of the difference in the mean iModulon activity level and compared it against the iModulon's log-normal distribution to determine a p-value. We performed this comparison (two-tailed) for a given pair of conditions across all iModulons at once and designated significance as FDR < 0.01. Only iModulons with a change in activity levels greater than 5 were considered significant and shown on the plot. Only one iModulon is significant, which was true for the aggregate and the individual comparisons: 'Uncharacterized-5'. It is likely that this iModulon is directly capturing the difference in the transcriptome. Encouragingly, it includes the *mexB* gene, which is the knockout of the PAO1(*ΔmexB*) strain.

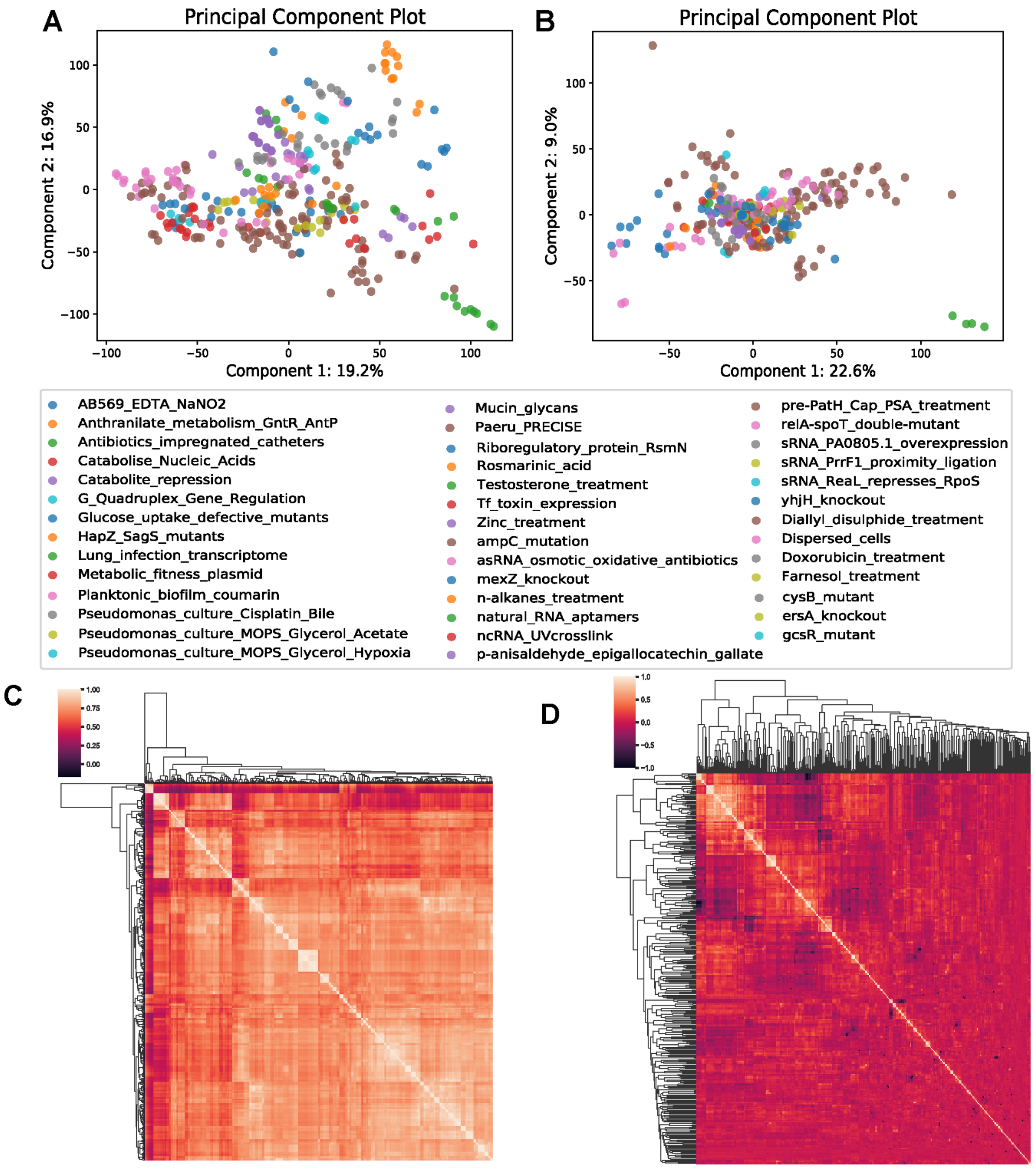

Supplementary Figure S1. Evaluation of the batch effect after the normalization. *The principal component plot shows the diversity of the samples used in the study. The color represents different projects used in the study. A) before normalization, b) after normalization. The clustermap shows the global correlations between one sample and all others along with the hierarchical clustering. C) before normalization, and D) after normalization.*

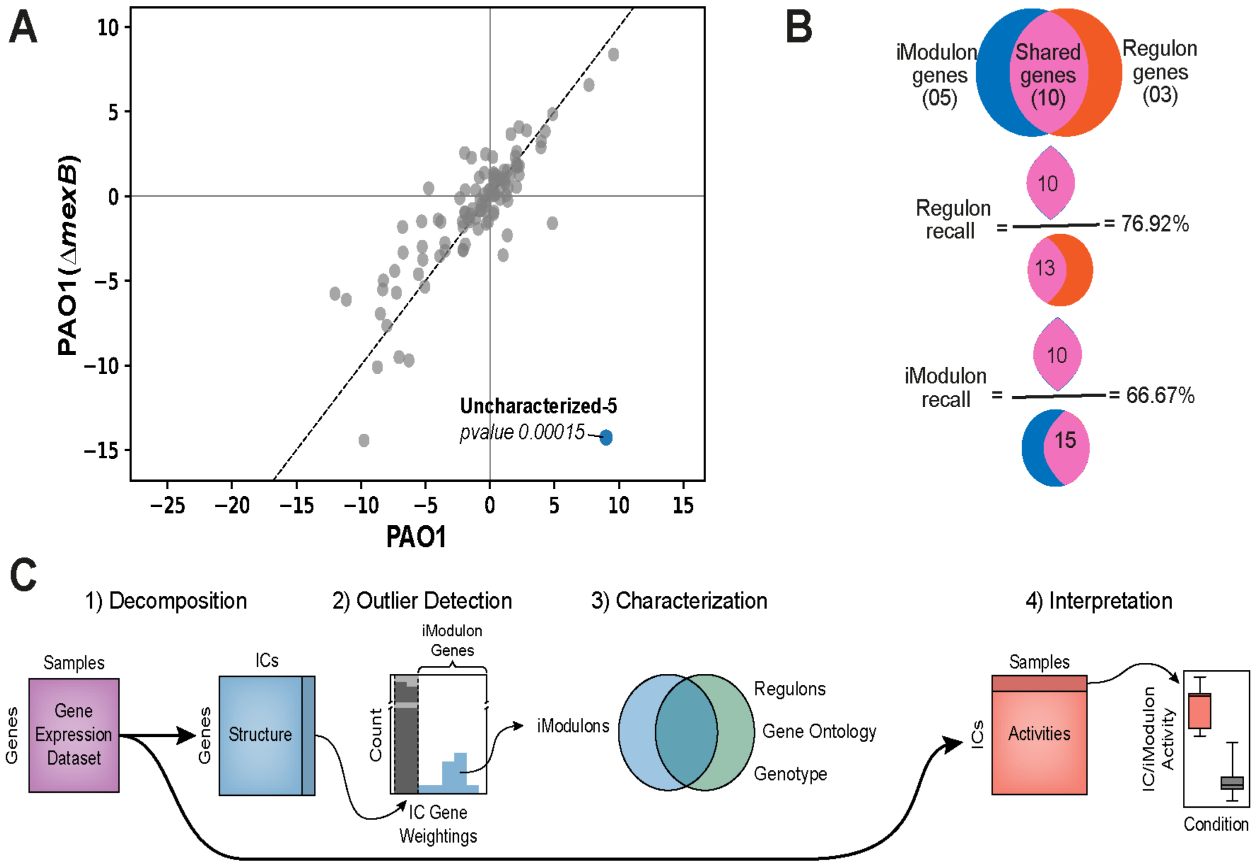

Supplementary Figure S2. Overview of the RNAseq data of Pseudomonas aeruginosa used in the study. *A) Differential activation analysis showing the difference in the activities (104 iModulons) of the samples in PAO1 and PAO1(ΔmexB). From the in-house generated samples, we plotted M9fructAA, M9GlcNAcAA, M9pyruvAA, M9sucAA, M9glucAA, and M9glucAA_NaCl to compare the iModulon activities in both the PAO1 and PAO1(ΔmexB). B) Schematic representation of the definitions of regulon recall and iModulon recall. C) Detailed overview of the methodology used in the study. It includes decomposition, outlier detection, characterization, and interpretation of the iModulons. (Adapted from Sastry et al. (1)).*

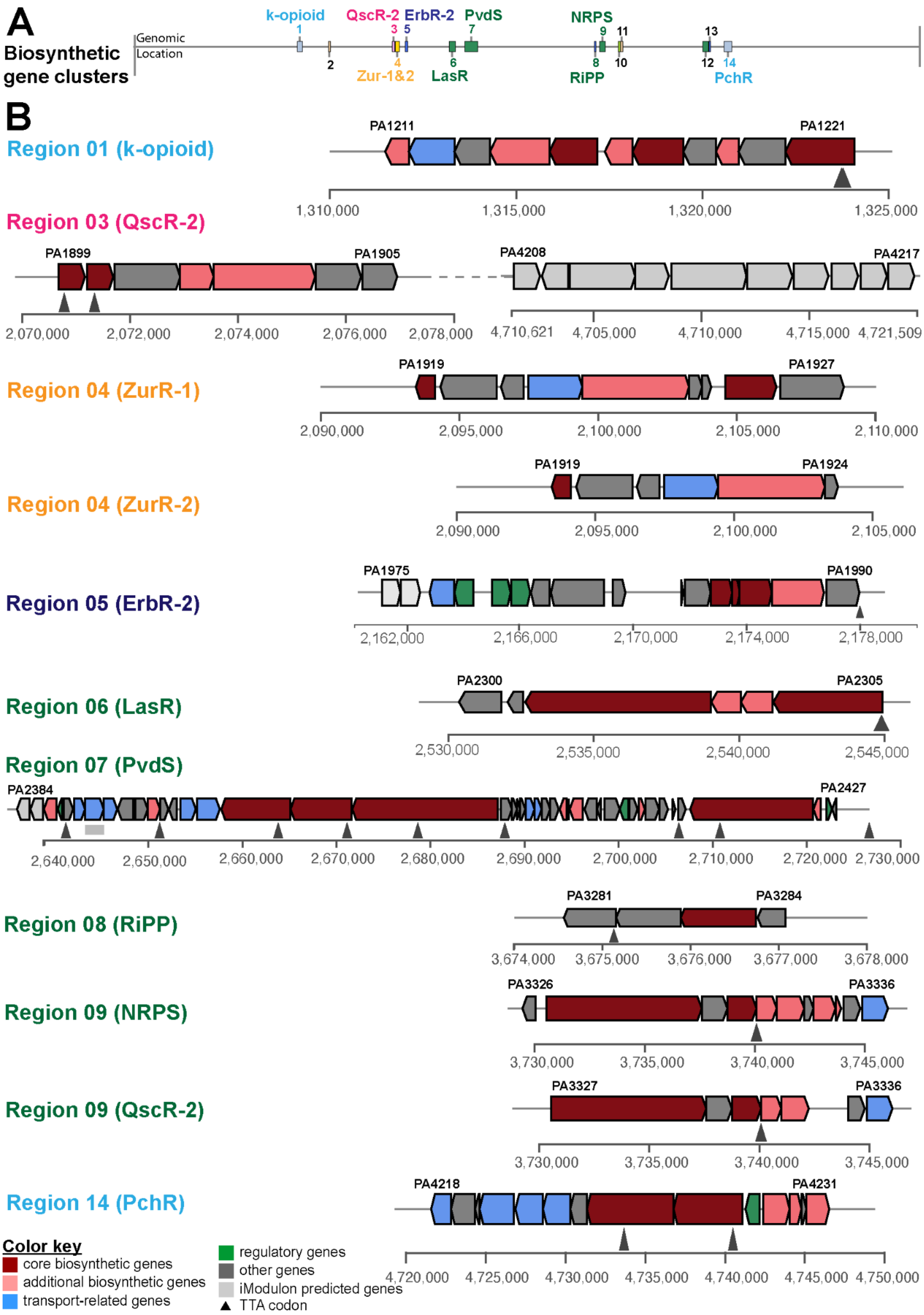
A
Biosynthetic gene clusters
Genomic Location
k-opioid
QscR-2
ErbR-2
PvdS
NRPS
Zur-1&2
LasR
RiPP
PchR
B
Region 01 (k-opioid)
PA1211
PA1221
1,310,000
1,315,000
1,320,000
1,325,000
Region 03 (QscR-2)
PA1899
PA1905
PA4208
PA4217
2,070,000
2,072,000
2,074,000
2,076,000
2,078,000
4,710,621
4,705,000
4,710,000
4,715,000
4,721,509
Region 04 (ZurR-1)
PA1919
PA1927
2,090,000
2,095,000
2,100,000
2,105,000
2,110,000
Region 04 (ZurR-2)
PA1919
PA1924
2,090,000
2,095,000
2,100,000
2,105,000
Region 05 (ErbR-2)
PA1975
PA1990
2,162,000
2,166,000
2,170,000
2,174,000
2,178,000
Region 06 (LasR)
PA2300
PA2305
2,530,000
2,535,000
2,540,000
2,545,000
Region 07 (PvdS)
PA2384
PA2427
2,640,000
2,650,000
2,660,000
2,670,000
2,680,000
2,690,000
2,700,000
2,710,000
2,720,000
2,730,000
Region 08 (RiPP)
PA3281
PA3284
3,674,000
3,675,000
3,676,000
3,677,000
3,678,000
Region 09 (NRPS)
PA3326
PA3336
3,730,000
3,735,000
3,740,000
3,745,000
Region 09 (QscR-2)
PA3327
PA3336
3,730,000
3,735,000
3,740,000
3,745,000
Region 14 (PchR)
PA4218
PA4231
4,720,000
4,725,000
4,730,000
4,735,000
4,740,000
4,745,000
4,750,000
Color key
core biosynthetic genes
additional biosynthetic genes
transport-related genes
regulatory genes
other genes
iModulon predicted genes
TTA codon

Supplementary Figure S3. Overview of the biosynthetic gene clusters (BGCs) of Pseudomonas aeruginosa predicted from our ICA based analysis. *A) Genomic location of the 14 BGCs predicted from the antiSMASH software. B) structure of 11 BGCs defined by our ICA based pipeline*

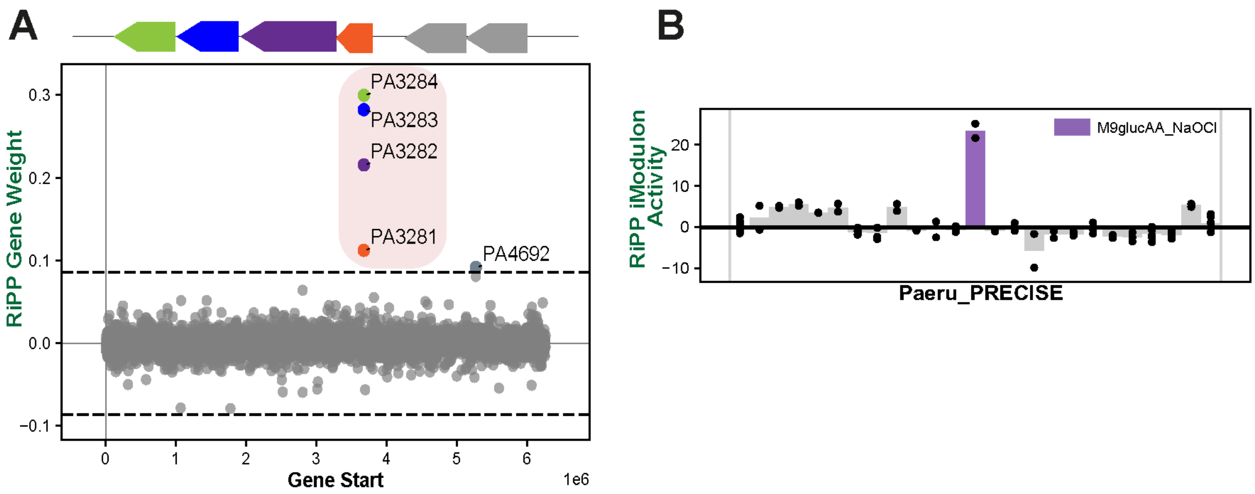

Supplementary Figure S4. Novel RiPP iModulon. *A) Scatter plot showing the gene weights of the novel RiPP iModulon, the color depicts the COG categories. B) Activity plot of the conditions expressed in RiPP iModulon in the Paeru_PRECISE*

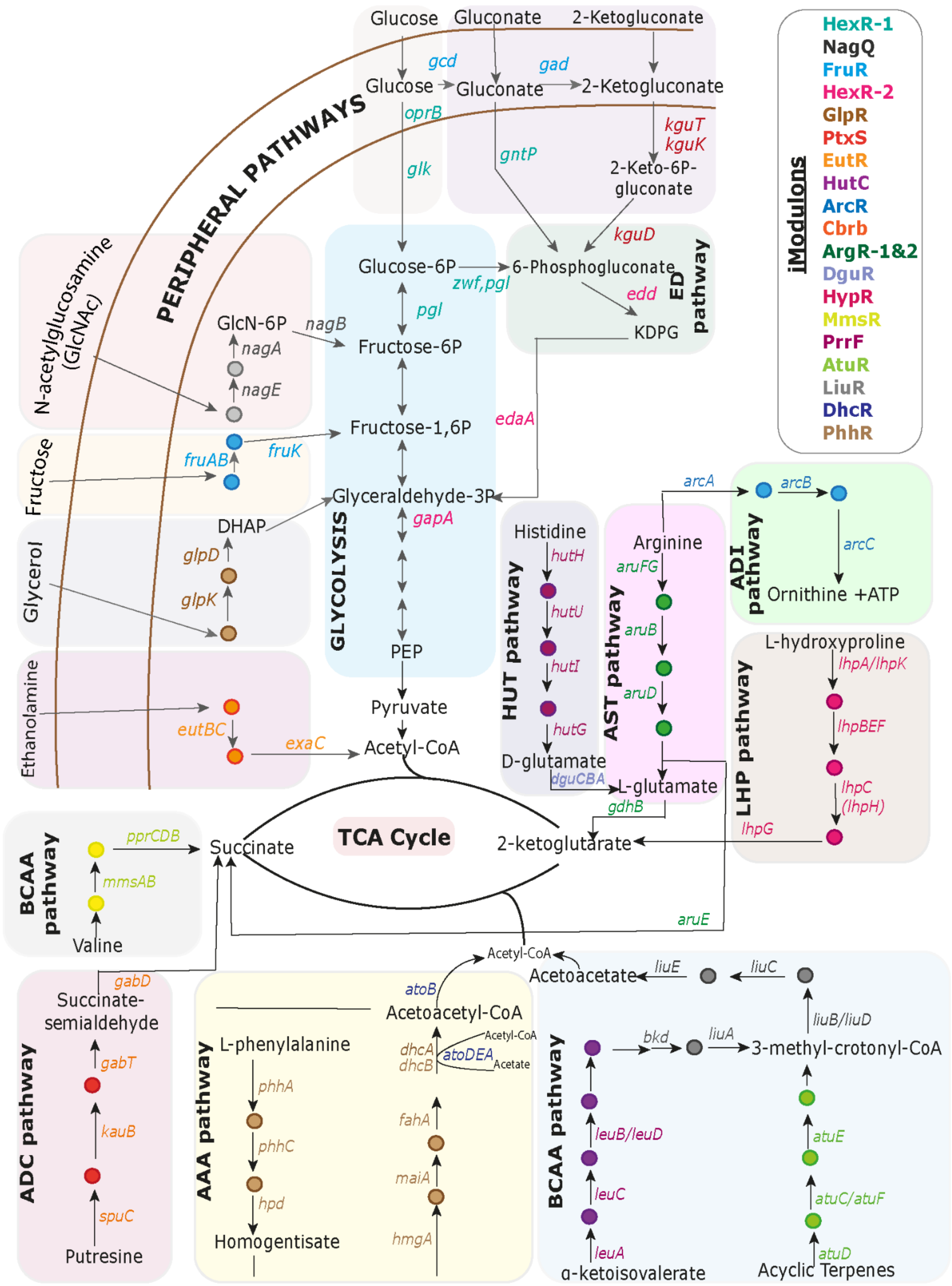
PERIPHERAL PATHWAYS
Glucose
Gluconate
2-Ketogluconate
gcd
gad
Glucose
Gluconate
2-Ketogluconate
oprB
kguT
kguK
glk
gntP
2-Keto-6P-gluconate
kguD
Glucose-6P
6-Phosphogluconate
zwf,pgl
edd
ED pathway
KDPG
pgl
N-acetylglucosamine (GlcNAc)
GlcN-6P
nagB
nagA
nagE
Fructose-6P
Fructose-1,6P
edaA
Fructose
fruAB
fruK
Glyceraldehyde-3P
gapA
DHAP
glpD
glpK
Glycerol
GLYCOLYSIS
PEP
Pyruvate
Ethanolamine
eutBC
exaC
Acetyl-CoA
Histidine
hutH
hutU
hutI
hutG
HUT pathway
D-glutamate
dguCBA
Arginine
aruFG
aruB
aruD
AST pathway
L-glutamate
gdhB
arcA
arcB
arcC
ADI pathway
Ornithine +ATP
L-hydroxyproline
lhpA/lhpK
lhpBEF
lhpC (lhpH)
lhpG
LHP pathway
iModulons
HexR-1
NagQ
FruR
HexR-2
GlpR
PtxS
EutR
HutC
ArcR
Cbrb
ArgR-1&2
DguR
HypR
MmsR
PrrF
AtuR
LiuR
DhcR
PhhR
TCA Cycle
Succinate
2-ketoglutarate
pprCDB
mmsAB
Valine
BCAA pathway
aruE
Acetyl-CoA
Acetoacetate
liuE
liuC
gabD
Succinate-semialdehyde
gabT
kauB
spuC
Putresine
ADC pathway
atoB
Acetoacetyl-CoA
Acetyl-CoA
Acetate
atoDEA
dhcA
dhcB
L-phenylalanine
phhA
phhC
hpd
Homogentisate
fahA
maiA
hmgA
AAA pathway
liuB/liuD
bkd
liuA
3-methyl-crotonyl-CoA
leuB/leuD
leuC
leuA
α-ketoisovalerate
atuE
atuC/atuF
atuD
Acyclic Terpenes
BCAA pathway

Supplementary Figure S5. *Graphical representation of the iModulons predicted and mapped as carbon and amino acid metabolism pathways. The carbon metabolism pathways are ED pathway, glycolysis, and peripheral pathways. While the amino acid metabolism pathways includes the branched chain amino acid (BCAA), aromatic amino acid (AAA), histidine utilization (HUT), arginine decarboxylase (ADC), arginine succinyltransferase (AST), arginine deiminase (ADI), and L-hydroxyproline (LHP) pathways*

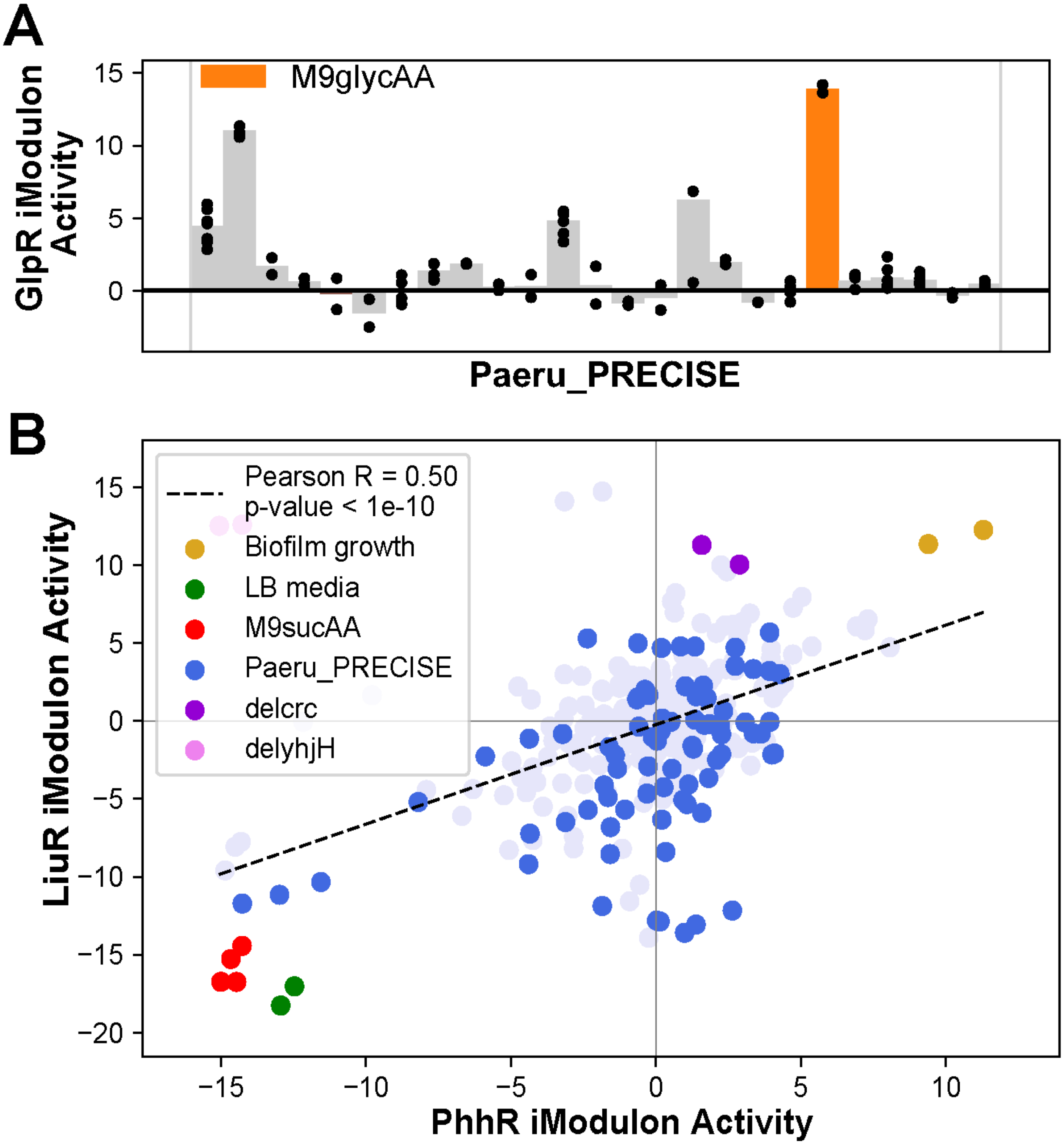

Supplementary Figure S6*. *iModulons related to the Carbon metabolism and the Amino Acid/Nucleotide metabolism. *A) Activity plot of the conditions expressed in GlpR iModulons in the Paeru_PRECISE; where M9glyAA is the samples collected in M9 media with glycerol and amino acids. B) Scatter plot showing the correlation between the BCAA pathways iModulons i.e. LiuR and PhhR with the PCC of 0.50; where M9sucAA is the samples collected in M9 media with sucrose and amino acids.*

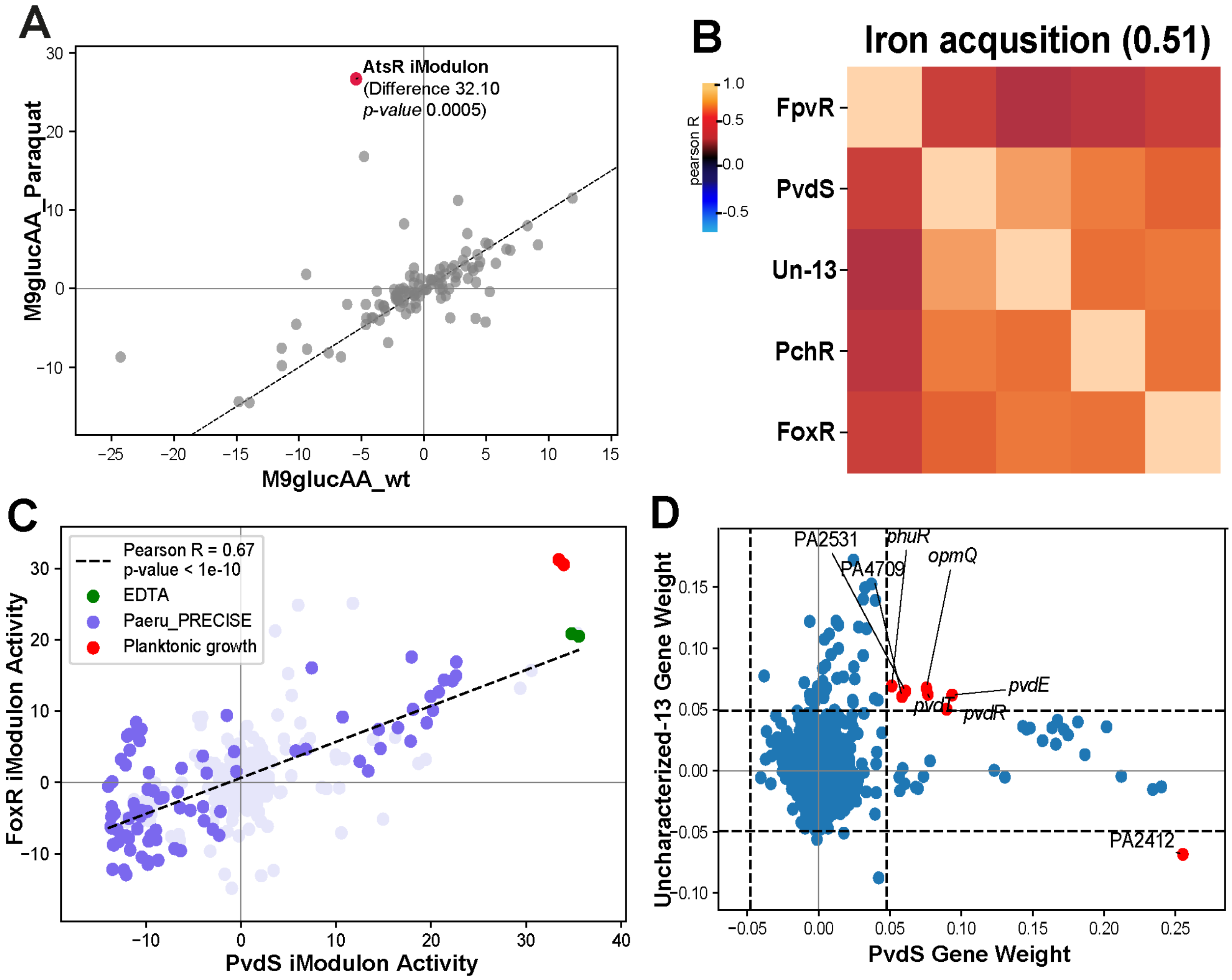

Supplementary Figure S7*. *Activity clustering of the iModulons among the P. aeruginosa. *A) Differential iModulon activity (DIMA) plot of the M9glucAA and Paraquat v/s the control condition showing the upregulation of the AtsR iModulon. B) Iron acquisition cluster with iModulons like FpvR, PvdS, Uncharacterized-13, PchR, and FoxR grouped with silhouette score of 0.51. C) The scatter plot shows the correlation between the FoxR and PvdS iModulons with PCC of 0.67. Both the iModulons show high activity in the EDTA and planktonic form of growth in P. aeruginosa. D) Scatter plot showing the gene weights between the Uncharacterized-13 and the PvdS iModulons. The red colored genes are common between both iModulons.*

References

1. Sastry,A.V., Hu,A., Heckmann,D., Poudel,S., Kavvas,E. and Palsson,B.O. (2021) Independent component analysis recovers consistent regulatory signals from disparate datasets. *PLoS Comput. Biol.*, 17, e1008647.

2. Wang,W., Tan,H., Sun,M., Han,Y., Chen,W., Qiu,S., Zheng,K., Wei,G. and Ni,T. (2021) Independent component analysis based gene co-expression network inference (ICAnet) to decipher functional modules for better single-cell clustering and batch integration. *Nucleic Acids Res.*, 49, e54.

3. Deng,K., Zhao,F., Rong,Z., Cao,L., Zhang,L., Li,K., Hou,Y. and Zhu,Z.-J. (2021) WaveICA 2.0: a novel batch effect removal method for untargeted metabolomics data without using batch information. *Metabolomics*, 17, 87.

4. Nazarov,P.V., Wienecke-Baldacchino,A.K., Zinovyev,A., Czerwińska,U., Muller,A., Nashan,D., Dittmar,G., Azuaje,F. and Kreis,S. (2019) Deconvolution of transcriptomes and miRNomes by independent component analysis provides insights into biological processes and clinical outcomes of melanoma patients. *BMC Med. Genomics*, 12, 132.

5. Sastry,A.V., Hu,A., Heckmann,D., Poudel,S., Kavvas,E. and Palsson,B.O. (2021) Independent component analysis recovers consistent regulatory signals from disparate datasets. *PLoS Comput. Biol.*, 17, e1008647.

6. Sastry,A.V., Gao,Y., Szubin,R., Hefner,Y., Xu,S., Kim,D., Choudhary,K.S., Yang,L., King,Z.A. and Palsson,B.O. (2019) The Escherichia coli transcriptome mostly consists of independently regulated modules. *Nat. Commun.*, 10, 5536.

7. Altschul,S.F., Gish,W., Miller,W., Myers,E.W. and Lipman,D.J. (1990) Basic local alignment search tool. *J. Mol. Biol.*, 215, 403–410.

8. Sastry,A.V., Poudel,S., Rychel,K., Yoo,R., Lamoureux,C.R., Chauhan,S., Haiman,Z.B., Al Bulushi,T., Seif,Y. and Palsson,B.O. Mining all publicly available expression data to compute dynamic microbial transcriptional regulatory networks. 10.1101/2021.07.01.450581.
